# A narrative systematic review of snakebite risk factors in Kenya, incorporating grey and academic literature

**DOI:** 10.1371/journal.pgph.0006344

**Published:** 2026-10-21

**Authors:** Eleanor Collinson, Rikki Gumbs, Shelley Lees, Alex Bowmer

**Affiliations:** 1 Department of Public Health and Policy, London School of Hygiene and Tropical Medicine, London, United Kingdom; 2 Institute of Zoology, Zoological Society of London, London, United Kingdom; The University of Georgia School of Health Sciences, GEORGIA

## Abstract

Snakebite is a neglected tropical disease, the drivers of which remain poorly understood and controlled in many countries. Incorporating grey literature has been recommended by previous academic publications on snakebite, due to the limited studies available globally, to provide a more accurate understanding of burden. In this review we aimed to identify and better understand risk factors that are associated with increased risk of snakebite, specifically across Kenya, using both grey and academic literature sources. We appraised academic studies using the Mixed Methods Appraisal Tool, while grey literature was assessed using the Authority, Accuracy, Coverage, Date and Significance guidelines. This systematic review synthesised evidence from 16 eligible peer-reviewed studies and 4 grey literature sources (1994–2025) examining determinants of snakebite risk and outcomes across Kenya. Given the limited number of eligible studies and heterogeneity of study types we conducted a thematic narrative synthesis. A total of 25 variables clustered into four domains were assessed, spanning human demographic factors, and environmental exposures. This study concurred with established literature on factors such as rurality, poverty and farming increasing snakebite risk, but also highlights discrepancies with global data. Several studies reported no seasonal variation or more bites in the dry season; bites were reported to occur equally in the day and the night in some studies; and no significant gender difference was observed in several reports. Overall findings converged on a consistent picture, snakebite envenoming in Kenya is concentrated among poorer, rural populations, and is more consistently linked to livelihoods and housing conditions, than seasonality or temporality. Actual burden and species responsible for bites remain poorly understood.

## Introduction

Venomous snakebites are currently the only World Health Organisation (WHO) listed non-infectious neglected tropical disease (NTD) [1,2]. Snakebite poses a significant global public health risk particularly in the global south, while simultaneously receiving relatively little investment in research, prevention, and treatment compared to other diseases with similar impact [2–4]. Snakebite envenoming results in an estimated 80,000–140,000 reported deaths, and ~400,000 permanent disabilities worldwide annually [2,5–8]. These figures are likely an underestimation as data is provided by health centres, and there is a growing consensus that Health Centre data fail to capture the true number of snakebite incidents worldwide [9–12]. This is likely due to long travel distances and the financial burden of accessing treatment in rural communities in Africa and Asia particularly [9,11,13–19].

As well as having higher levels of underreporting, communities living in remote, tropical regions are also thought to bear the highest burden from snakebites [10,13,19]. This is evidenced by numerous household and community surveys carried out in regions in Iran [20], Nepal [14,21,22], Mozambique [11], and Sri Lanka [9,10,23], which found that the number of snakebites reported by community members were 3–5 times higher than the official WHO reports of snakebites reported by that region [9,10,14,20,21].

Global incidence data, though limited, appears to suggest that snakebite burden is not primarily driven by the diversity of snakes, but by other factors possibly of socioeconomic or environmental nature [14,24–26]. Supporting this, though Australia has the highest relative diversity of venomous snakes (100 venomous species out of 170 species total), there are an average of two snake-related deaths annually [27,28], compared to the suspected 4,000 snakebite related deaths per year in Kenya [3,13].

Kenya is home to 66 species of venomous snakes [29]. However not all species pose a danger to human health. The WHO categorises certain species of venomous snakes as “medically significant”, referring to snake species that pose a significant risk to human health due to their venom LD_50_ (lethal dose 50% of a tested population, commonly used to measure acute toxicity) and potential for causing snakebites [2,6]. Venomous species that are deemed medically important in Kenya primarily include species belonging to the Viperidae and Elapidae families [2,6].

Bite incidence in Kenya is reported unevenly across the country, with limited data available to indicate areas of high incidence and the risk factors driving this [30–32]. To better understand this heterogeneity this review narratively synthesises named drivers of snakebite in Kenya from eligible studies. In response to previous studies suggesting the incorporation of grey literature to bridge the data gap [5,8,24], this systematic review incorporates both academic and grey literature sources to determine common risk factors associated with snakebite across Kenya. We hypothesise that regional variation in snakebite incidence in Kenya may be partly explained by differences between arid and semi-arid lands (ASALs) and non-ASAL areas, which differ in environmental conditions, and that variation in socioeconomic and climactic variables may further influence snakebite.

## Materials and methods

This review was conducted in accordance with the Preferred Reporting Items for Systematic Reviews and Meta-Analyses (PRISMA) checklist (see S1 PRISMA Checklist) [33]. The review protocol described here was registered with PROSPERO (registration number CRD420251102334 on 11 July, 2025), prior to completion of systematic literature search.

### Search strategy

A systematic search was conducted between October 2024 and September 2025 across four academic databases: PubMed, Scopus, Senate House Library and Google scholar, with a last search on September 30th, 2025. The following search terms and medical subject heading (MeSH) terms were used across all databases: “snake”; “snakes”; “risk”; “snakebite”; “snake bite”; “snake-bite”; “envenomation”; “venom”; “venomous”; “Kenya”; “human-wildlife conflict”; “incidence”. These terms were joined using the Boolean operator OR/ AND/ IN. Both groups were then combined with the operator AND. An asterisk (‘*’) was used to search for variation in the ending of search terms. No language restrictions were placed during the search.

### Study selection

All retrieved records were exported to Zotero and duplicates were removed. The initial search, title and abstract screening, full-text assessment, and eligibility of studies (Table 1) were conducted by two reviewers ([AB] and [SL]). Titles and abstracts were screened to ensure they lined up with the inclusion/exclusion criteria, then full studies were screened. Any disagreements were resolved by involving a third reviewer ([EC]). Due to the limited number of studies available focused on Kenya there was no strict measures on study design or cutoff date for age of articles. For grey literature to be considered from a reputable source the authors needed to be either established herpetologists, Non-Government organisations (NGOs) with a specialism in snakebite, or government databases and reports. While no language restrictions were placed on sources searched, only English publications met the inclusion criteria, likely due to the main publishing language of Kenya being English, see supplementary S1 Table for further information.

**Table 1:** Inclusion and Exclusion criteria for selected texts.

Factor	Inclusion	Exclusion
**Population**	Humans of any age exposed to venomous snakebite in Kenya.	Captive animal studies, studies conducted or about exotic pets. Papers focused on after care of bites.
**Location**	Kenya as a study site explicitly mentioned in article	Studies where Kenya is not included or global reviews where Kenya is not specifically mentioned or data from Kenya not explicitly used.
**Species**	Venomous Snake Species found in Kenya	Snake species not found in Kenya or the article doesn’t separate snake/snakebite risk from other poisons or wildlife.
**Exposure/Risk factors**	Environmental, ecological, socioeconomic, behavioural or health-service determinants of snakebite incidence, human determinants, factors impacting outcome of bite.	Laboratory toxin studies; antivenom pharmacology; no risk factors mentioned. Only outcome mentioned but no risk factors in bite occurrence.
**Outcomes**	(i) Incidence, prevalence or mortality of snakebite; (ii) any quantified association between a candidate driver and bite occurrence or outcome	Purely descriptive case reports with no associated risk-factor data.
**Source**	Peer reviewed or a source of authority such as government or NGO, or recognised journal published	Not from a verifiable authority or academic paper. Other systematic reviews, data needs to be from primary source.

Table 1 Study Eligibility Criteria: Detailed inclusion and exclusion criteria used during title/abstract and full-text screening see Fig 1, for screening process.

**Fig 1:**
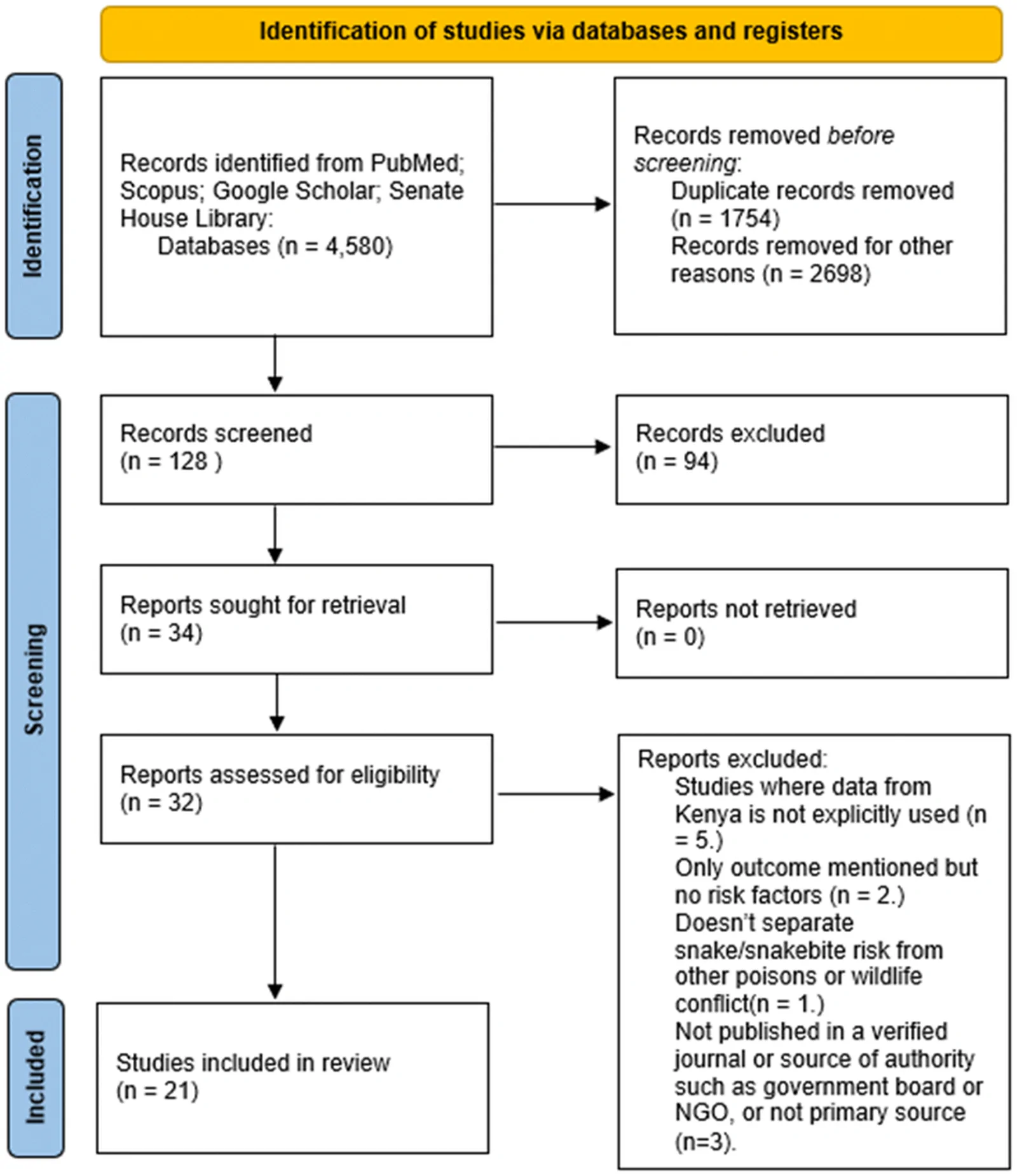
PRISMA flowchart of study selection process. The chart outlines the exclusion criteria applied at each stage of screening. The reasons for article exclusion during the initial screening phase correspond to all the exclusion criteria specified in Table 1.

### Eligibility criteria

### Data extraction and outcome measure

The primary outcome measured was named risk factors associated with direct incidence, or increased risk, of snakebite. All quantitative data reported risk were extracted from the articles. The information on the eligible studies was collected in a standardised data extraction table developed in Microsoft Excel (Microsoft 365) by the researcher EC and exported to RStudio (version 4.3.2), extracted data tables were reviewed by RG. The following data fields were collected: publication identifiers; title; geographic scope; age of study; study design; population demographics; location; number of people presenting snakebite if documented; species of snakes; and environmental risk factors. Any discrepancies in data extraction were discussed and missing data is discussed in the limitations of studies section.

### Risk of bias and quality assessment of articles

Due to the variety of studies, we appraised methodological quality of academic papers using the Mixed Methods Appraisal Tool (MMAT, 2018) [34]. The checklist comprises eight questions which can be answered “yes”, “no” or “unclear” and address methodological quality and risk of bias. Following MMAT guidance, we report criterion-level ratings, whereas the AACODS checklist (Authority, Accuracy, Coverage, Objectivity, Date, Significance) which is comprised of six questions addressing the quality and academic authority of the source was applied to grey literature [35]. AACODS appraisal tool for quality assessment and risk of bias. For an explanation of each category and full guidelines on AACODS and MMAT appraisal see supplementary Tables 3 and 4. We excluded low scoring studies (<4 for the MMAT) in line with other systematic reviews. Further details can be found as supporting information. Two reviewers (AB and SL) independently assessed each study according to the relevant domain (qualitative; randomised; non-randomised; quantitative descriptive; mixed methods). Discrepancies were resolved by third reviewer (EC).

### Data synthesis

Due to the heterogeneity in study designs and the inclusion of grey literature, we conducted a narrative synthesis following SWiM guidance [36]. All types of snakebite outcomes were considered together. Factors were coded 1 if they were associated with increased snakebite risk, 0 if the factor was explicitly stated to not increase risk above background risk levels, in the population and NA if not mentioned. Due to limited suitable studies factors that appeared in 3 more sources were considered common variables and were included in the comparisons, in line with previous papers on snakebite risk variables [37] associated variables were grouped as follows:

1) **Demographic & Socioeconomic Factors:** Gender distributions, no significant sex difference was counted if there was less than 5% difference between males and females reported (aka 47.9: 52.1 either direction), this figure was chosen to reflect the minimum 0.05 confidence level of a significant P-value; Age was classified as under 18 years, or 18 years and older. Where reported, age was extracted narratively from each study. Because individual studies could describe multiple snakebite cases, both age categories could be represented within a single paper. Indicators such as poverty, housing type, employment/unemployment levels, barriers to health care and other markers of socioeconomic status (SES) were grouped under SES/poverty. If not explicitly labelled as poverty in text, indicators of low SES were applied from Oxford Poverty and Human Development Initiative (OPHI) guidelines on Multiple Poverty Indicators (MPI) calculation, such as housing structure, sanitation, basic public services and infrastructure indicators [38].
2) **Geographic & Environmental Factors:** Land cover type if mentioned such as savannah (also including variations and terminology such as “bush” and “savana”), arid/semi-arid landscape, urban, or cropland; urban/rural setting; did the bite occur in or outside of the home. A result for one factor did not rule out a result for another factor unless explicitly stated, for example bite risk could be cited as occurring in cropland AND savannah and both categories would be scored 1. A category would only be scored 0 if explicitly stated not to be associated with bite risk. This scoring method was kept consistent for all factor scoring across categories.
3) **Occupational and Activity Related Risk Factors:** Occupation type; activity at time of bite such as walking, herding, sleeping, playing, farming, collecting firewood. These groups were placed together as they are often overlapping, aka farmers are often reported farming or walking at the time of bite. Outside and Inside were taken from text if explicitly stated, or if the risk associated activity could be definitively tied to being inside or outside, such as “herding” is exclusively and activity done outside the home.
4) **Temporal and Climactic factors associated with snakebite risk:** Rainy season, dry season or no seasonal difference if reported; Time of day of incidence (aka whether bite occurred at night or in the day, if time was specified between 6am and 6pm was counted as “Day Time” as these are the hours of daylight and 6pm to 6am were counted as “Night Time”. Both day and night equally was counted if there was less than 5% difference between day and night reported (aka 47.9: 52.1 either direction), this figure was chosen to reflect the minimum 0.05 confidence level of a significant P-value. Day/night classification was sometimes narratively inferred from activity when appropriate if not specifically mentioned, for example if bites were reported to happen during “the dark” these bites were assumed to have happened between 18:00 – 06:00 hours (see supplementary S1 Data for full classification list of activity- time inferences). However these cases were limited to explicitly time bound activities.

Each study was classified into one of two regional categories: ASAL (arid and semi-arid lands) or MIX (studies spanning both ASAL and non-ASAL counties). The categorisation of ASAL counties was based off of average annual rainfall received [39]. Because no studies were available representing the non-ASAL category, comparisons were limited to ASAL and MIX groups. In the MIX studies for many it was often not possible to determine which county the data had come from and as such, due to the small sample size of available studies, results were pooled into ASAL and MIX only. A full table of each county covered in each study can be found in supplementary S1 Data.

For each risk factor, if the factor showed mixed support and was not clearly positively associated with increased snakebite risk then the factor was examined geographically to see if the mixed support could be explained by environmental factors. To do this the proportion of studies reporting that variable was calculated separately for ASAL and MIX studies proportions were computed for descriptive comparison. Results were rounded to two decimal places and interpreted cautiously in light of limited study numbers (most variables were reported in fewer than ten studies per category). Fisher’s exact test was used to compare proportions between groups due to the small sample size and sparse data structure and pairwise tests were run to explore correlations of variables (S1 Fig and S2 Table).

For each prespecified risk factor we coded direction of effect in Rstudio (version 4.3.2) (increased risk = 1; decreased risk = 0; not assessed = NA). Missing or unspecified information (*NA)* was excluded from denominator totals when calculating proportions, data for coded studies can be found in S5 Table. We summarized the proportion of studies indicating increased risk among those that reported the factor. Binary data for each demographic variable were averaged across all studies (excluding *NA* values) to obtain the percentage of studies that reported each factor. Data processing, statistical analyses and visualisation were conducted in R using the tidyverse [40], janitor [41], broom [42], grid [43], dplyr [44], tibble [45], pheatmap [46] and ggplot2 (version 4.0.2) [47].

## Results

### Prisma flow diagram

An initial search across databases returned 4,580 hits. After titles were screened a total of 128 studies were identified across included databases. Following PRISMA guidelines [33] after duplicates were removed and title and abstract screening a total of 34 studies were included for full text screening (Fig 1). After a full text screen a total of 21 academic and grey studies that had data pertaining specifically to snakebite risk in Kenya were included for quality and bias assessment. A total of 23 of Kenya’s 47 counties were represented in the literature, and four nationwide studies were included.

### Quality and risk of bias assessment

Following the quality and risk of bias assessment from AACODS (Table 2) and the MMAT [34] (Table 3) (find scoring guide in S3 and S4 Table’s) the results of included literature can be found below. As the MMAT scoring system is tailored to the different study designs a full breakdown of the MMAT criteria can be found in supplementary S Table. Scoring criteria for papers indicated that overall quality of academic and grey literature was high. Common strengths of included papers were clear and appropriate approaches to answering the research questions and study design. Due to the nature of the tailored MMAT scoring system there was not any consistent frequent limitations across papers. Many studies met the inclusion criteria and were robust in methodological quality, however we excluded studies meeting fewer than half of applicable criteria (e.g., Coombs et al1997, Table 3).

**Table 2:** AACODS appraisal tool results.

Source	Authority	Accuracy	Coverage	Objectivity	Date	Significance	Include
Health Action International 2019: *Fact Sheet SNAKEBITE INCIDENTS,* *RESPONSE & ANTIVENOM SUPPLY (KILIFI COUNTY, KENYA).*	Y	Y	Y	Y	Y	Y	Y
Health Action International. (2021). *Snakebite in Kenya: Evidence from the field, research data from 2019–2020*.	Y	Y	Y	Y	Y	Y	Y
Ministry of Health & Kenya Medical Association. (2019). *Guidelines for prevention, diagnosis, and management of snakebite envenoming in Kenya*.	Y	Y	Y	Y	Y	Y	Y
Spawls, S., & Branch, B. (2020). *The dangerous snakes of Africa*.	Y	Y	Y	Y	Y	Y	Y

Table 2 Quality assessment of included studies (n = 4) Y indicates “Yes, category satisfied, “N” shorthand for no indicating insufficient evidence to pass on that category. Full details of scoring for AACODS can be found in S3 Table.

**Table 3:** MMAT appraisal tool for mixed method studies.

Study (Author, Year, DOI)	Study Category	S1	S2	C1.1/3.1/4.1/5.1	C1.2/3.2/4.2/5.2	C1.3/3.3/4.3/5.3	C1.4/ 3.4/4.4/ 5.4	C1.5/ 3.5/4.5/5.5
Abouyannis M, et al. 2023. *PLoS Negl Trop Dis.* https://doi.org/10.1371/journal.pntd.0010987	Quantitative Non-Randomized	yes	yes	yes	yes	yes	yes	yes
Barnes K, et al. 2021. *Toxicon X.* https://doi.org/10.1016/j.toxcx.2021.100078	Qualitative Research	yes	yes	yes	yes	yes	yes	yes
Coombs MD, et al. 1997. *Trans R Soc Trop Med Hyg.* https://doi.org/10.1016/S0035-9203(97)90091-2	Quantitative Descriptive Studies	no	yes	no	no	no	cant tell	cant tell
Kihiko DK. 2013. *Ann Afr Surg.* https://doi.org/10.4314/aas.v10i1	Quantitative Descriptive Studies	yes	yes	yes	yes	yes	yes	yes
Kung’u PN, et al. 2023. *Int Health.* https://doi.org/10.1093/inthealth/ihac043	Quantitative Descriptive Studies	yes	yes	yes	yes	no	yes	yes
Larson PS, et al. 2022. *Trop Med Health.* https://doi.org/10.1186/s41182-022-00421-8	Mixed Methods Studies	yes	yes	yes	yes	yes	yes	yes
Mukeka JM, et al. 2019. *Glob Ecol Conserv.* https://doi.org/10.1016/j.gecco.2019.e00620	Quantitative Descriptive Studies	yes	yes	yes	yes	yes	yes	yes
Mukeka JM, et al. 2019. *Int J Biodivers Conserv.* https://doi.org/10.5897/IJBC2019.1278	Quantitative Descriptive Studies	yes	yes	yes	yes	yes	yes	yes
Okumu MO, et al. 2018. *Pan Afr Med J.* https://doi.org/10.11604/pamj.2018.29.217.15366	Quantitative Descriptive Studies	yes	yes	yes	unclear	no	no	yes
Okumu MO, et al. 2019. *F1000Res.* https://doi.org/10.12688/f1000research.20268.1	Quantitative Descriptive Studies	yes	yes	yes	yes	yes	yes	yes
Ooms GI, et al. 2021. *Am J Trop Med Hyg.* https://doi.org/10.4269/ajtmh.20-1078	Quantitative Descriptive Studies	yes	yes	yes	no	no	yes	yes
Ooms GI, et al. 2021. *Am J Trop Med Hyg.* https://doi.org/10.4269/ajtmh.21-0266	Quantitative Descriptive Studies	yes	yes	yes	yes	yes	yes	yes
Snow RW, et al. 1994. *Ann Trop Med Parasitol.* https://doi.org/10.1080/00034983.1994.11812919	Quantitative Descriptive Studies	no	Cant tell	yes	yes	yes	yes	yes
Tianyi FL, et al. 2024. *PLoS Negl Trop Dis.* https://doi.org/10.1371/journal.pntd.0011678	Quantitative Descriptive Studies	yes	yes	yes	yes	yes	yes	yes
Tianyi FL, et al. 2025. *PLoS Negl Trop Dis.* https://doi.org/10.1371/journal.pntd.0012845	Quantitative Descriptive Studies	yes	yes	yes	yes	yes	yes	yes
van Oirschot J, et al. 2021. *Trans R Soc Trop Med Hyg.* https://doi.org/10.1093/trstmh/trab059	Qualitative Research	yes	yes	yes	yes	yes	yes	yes
Wood L, et al. 2022. *PLoS Negl Trop Dis.* https://doi.org/10.1371/journal.pntd.0010579	Qualitative Research	yes	yes	yes	yes	yes	yes	yes

Table 3 MMAT appraisal tool for quality assessment and risk of bias has different questions for each section depending on what type of study is being assessed. Studies were marked “yes if they satisfied the criteria, “no” if they did not satisfy the criteria, and “unclear” if it was uncertain if criteria was met. For a list of each question and full guidelines on MMAT appraisal see S4 Table.

### Factors narratively associated with increasing snakebite risk

We identified 25 variables associated with either increased snakebite risk or neutral impact on snakebite risk (Fig 2), that appeared in the literature at least 3 separate times across studies. A table of all variables can be found in S1 Data. The strongest and most consistently reported risk factor was rurality (19/20, 95%) [29–31,48–63]. High support was also observed for general outdoor locations (Outside) as bite risk out of total studies (16/20, 80%) [30,31,48–58,60,61,63] and savannah regions.

**Fig 2:**
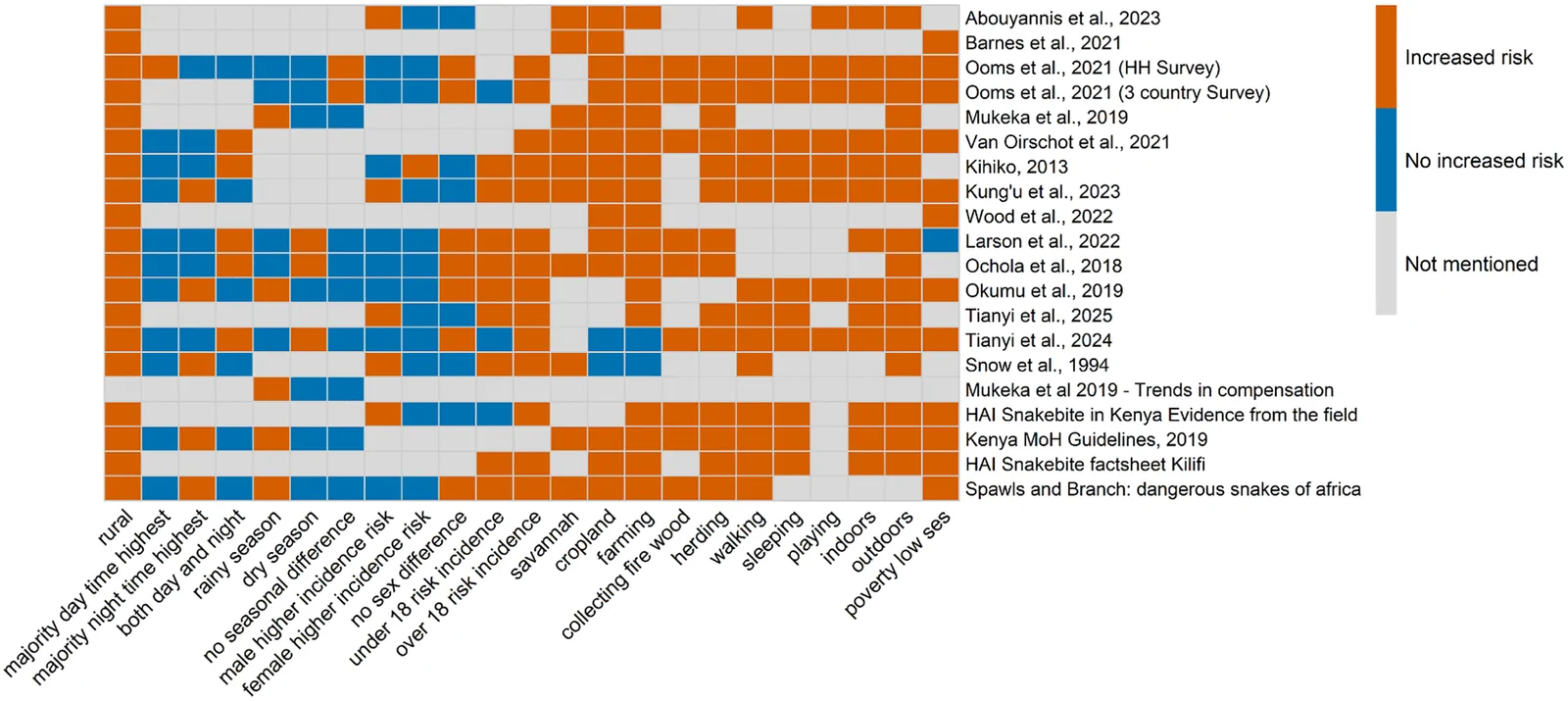
Heatmap of factors associated with increased snakebite risk. The heat map summarizes the most commonly mentioned risk factors, appearing 3 or more times in the literature, and which studies reported each prespecified factor as increasing snakebite risk (Orange = association with increased risk, Blue = not associated with increased risk above background level, grey = not mentioned NA). Vertical axis shows named risk factor and horizonal axis shows corresponding paper reference.

Bites occurring outside were frequently linked to activity factors such as farming (16/18, 88.88%) [29–31,48,50–55,57,58,60,61,63,64] within cropland habitat (14/16, 87.5%) [29,48,50–55,58–63]. However two studies, both from ASAL regions, did not find an increased risk during farming in cropland landscapes, and either reported an overall, unpredictable risk with relatively low levels of risk related to farming [49], or high snakebite risk to be associated with walking in grassy and cleared areas, not agricultural areas or farming [56]. Savannah, which was associated with pastoralism, was recorded as a risk prone habitat whenever included in studies narratives [29,48,49,51–53,58,60,62,63]. Both Walking and Herding were frequently cited as a risk factor specifically in the savannah [31,48,51,52,56,58,63]. Whereas Playing [48,49,51,53,55,57,61,63] was only reported when children were interviewed.

Bites were also reported to occur inside the home (13/20, 65%) [30,31,48–55,57,61,63], with one study reporting bite incidence to occur more frequently inside than outside the home [51]. Bites occurring inside of the house often occurred while sleeping (11/20, 55%) [30,31,48–55,57], and were frequently attributed to poverty metrics and building materials for the houses (e.g., earthen floors and structural holes facilitating snake entry [48–53,62,64]. In almost every paper where socioeconomic indicators of poverty were stated poverty metrics were noted as a risk factor for snakebite [29,30,49,51–55,57,61,62,64]. Only Larson et al., 2022 [50] did not find a strong correlation between poverty and snakebite risk, but found rurality to be the strongest indicator.

In contrast, several factors showed weaker support such as seasonality of bites: rainy season was associated with higher risk in 50% of studies (5/10) [29,52,57,60,65], but dry season (3/10, 30%) [49,50,58] and no seasonal difference (2/10, 20%) [55,61] were also commonly reported. Temporality of bites also showed variation in highest bite risk with night-time having higher reported risk in 5/11 (45.45%) of reports [29,51,52,56,57], however both day and night were reported to carry a similar bite risk in the other studies 5/11 (45.45%) [48–50,53,58], and one study reported a peak of bite incidence during the day time 1/11 (9.09%). There was also variation in the sex ratio of those affected, with men more frequently reported among snakebite cases in 5/13 studies (38.4%) [31,49,56,63], but the majority of studies reported no significant sex difference (7/13, 53.85%) [29,49,50,55,57,58,61]. This variation will be explored further below to examine if geographic factors can explain the variance observed in the results.

### Factors with mixed support of increasing snakebite risk

#### Demographic Factors Associated With Snakebite Risk in ASAL vs MIX Studies.

No significant difference between sexes was more frequently reported in MIX studies (5/6, 83.33%) [29,50,55,57,58], ASAL (2/7, 28.57%) [31,61] (Fig 3). Higher Male bite incidence was more commonly reported in ASAL studies (4/7, 66.14%) [31,51,56,63] than MIX (1/6, 16.67%) [30]. However, one study conducted in an ASAL setting reported a significantly higher number of snakebite cases among females (1/7, 14.28%) [48]. Though men appeared more frequently as higher risk in several reports, the actual percentage difference was often small, such as snow et al [56], the distribution was 57% men to 43% women. Only two studies reported a highly significant sex difference in snakebite incidence; one, conducted in an ASAL region, found that nearly twice as many women as men were bitten [48], and one was from a MIX study showing 2/3 of bite incidents involved men [30]. Under 18 risk was narratively reported frequently in both ASAL and MIX studies, though slightly more frequently in ASAL studies 5/6 (83.3%) [31,48,51,56] vs 4/6 MIX studies (66.7%) [29,50,57,58].}. Whereas over 18’s were a risk group in every study where age was accounted for (100%) [29–31,48,49,51,53–58,61].

**Fig 3:**
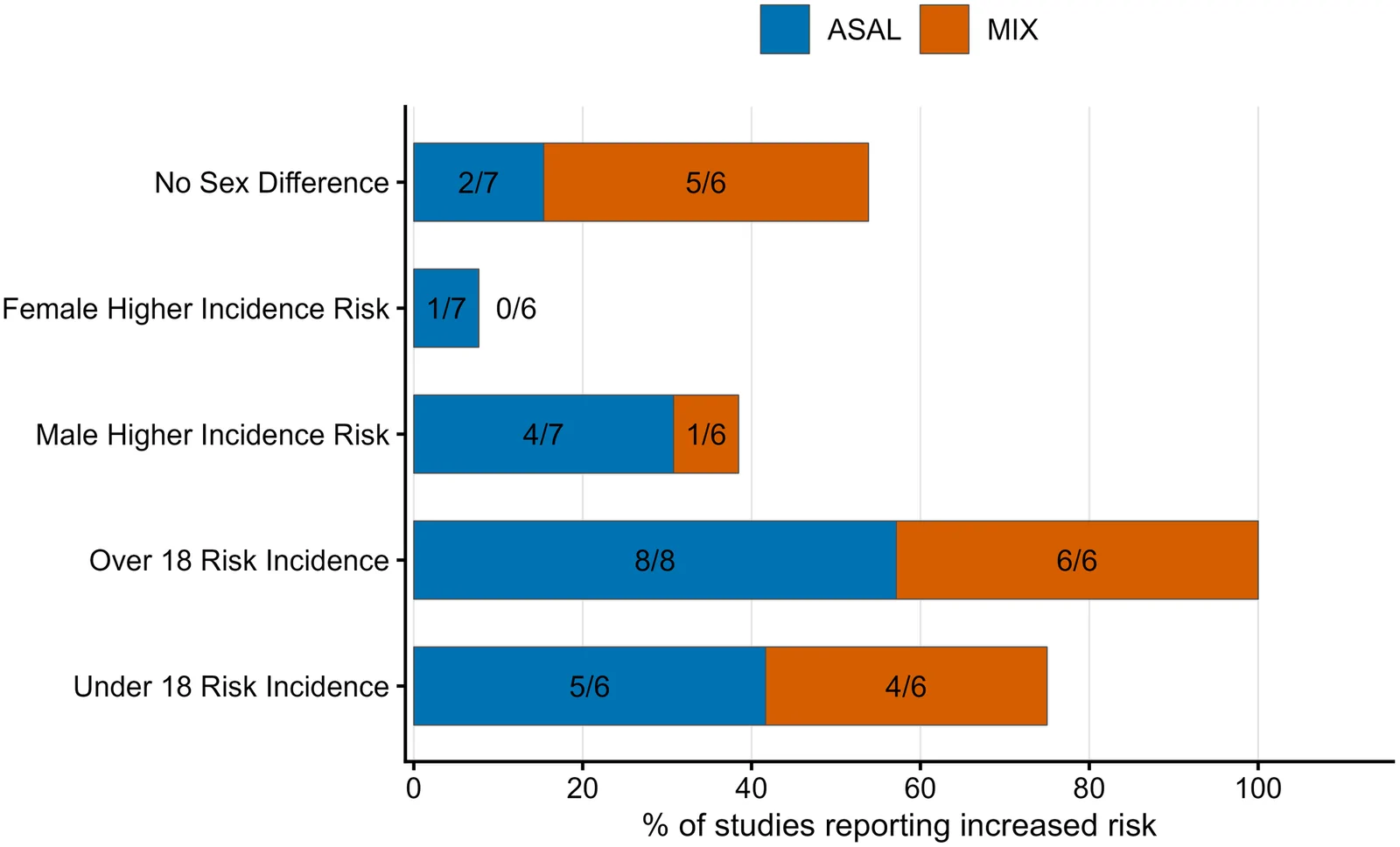
Proportion of studies reporting increased snakebite risk across demographic categories across ASAL and MIX studies. Horizontal stacked bars show the percentage of studies within each setting reporting elevated risk for each category. Labels indicate the number of studies supporting the finding out of the total number assessing that factor (e.g., 7/8). Categories include age (under 18 years; over 18 years), sex (male higher incidence; female higher incidence; no sex difference).

### Environmental factors associated with snakebite risk in ASAL vs MIX Studies

In line with literature from other countries a significant proportion of bites occur at night, often between 6 pm and 6 am, however this result was most frequently reported in MIX studies (3/5 (60%)) [29,52,57], vs 2/6 (33.33%) of ASAL studies (42,56) (Fig 4). Daytime was seen to also carry a risk of snakebite, particularly in an ASAL study by Larson et al,. 2022 where snakebite risk was recorded to be higher in the day than the night (ASAL: 1/6 (16.6%)) [51. In several studies across both ASAL and MIX settings snakebite risk was recorded as landscape level threat, rather than a time bound hazard, occurring almost equally in both day and night (ASAL 3/6 (50%) [48,49,53]; MIX 2/5 (40%) [50,58].

**Fig 4:**
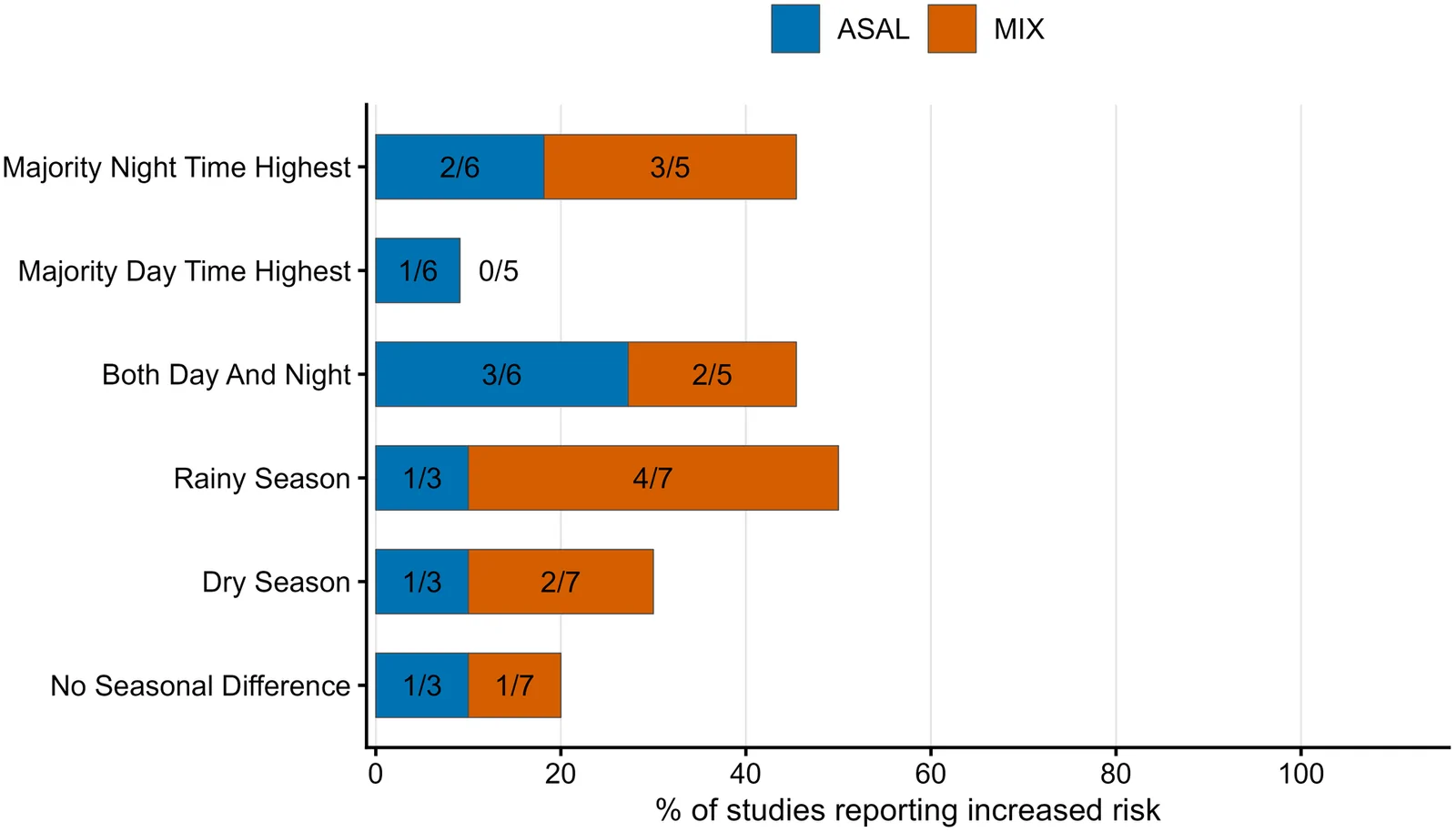
Proportion of studies reporting increased snakebite risk across Environmental categories in arid and semi-arid lands (ASAL - Blue) and mixed (MIX - Red) settings. Horizontal stacked bars show the percentage of studies within each setting reporting elevated risk for each category. Labels indicate the number of studies supporting the finding out of the total number assessing that factor.

When reporting seasonality of snakebite risk, the rainy season was reported to be associated with highest risk more frequently in MIX studies (4/7 (57%)) [29,52,58,65] than ASAL studies (1/3 (33.3%)) [60]. However, there was variation in seasonality reported across both ASAL and MIX studies, with the dry season reported to have higher incidence of snakebite risk in 1/3 (33.33%) of ASAL studies [49], and 2/7 (28.57%) MIX studies [50,58]. Other studies still reported no significant seasonal difference in bite risk, though this result was more frequently reported in ASAL studies (1/3 (33.33%)) [61] than MIX studies 1/7 (14.2%) [55].

## Discussion

### Summary of evidence

This systematic review synthesised evidence from 16 peer-reviewed studies and 4 grey literature sources (1994–2025) examining determinants of snakebite risk and outcomes across Kenya. Incorporating the use of grey literature has been recommended by previous reviews of snakebite due to the limited studies available globally [5,8,24]. A total of 26 variables clustered into four domains were assessed, spanning human demographic factors, and environmental exposures. Given the limited number of eligible studies and heterogeneity of study types we conducted a thematic narrative synthesis. The included studies were conducted across multiple counties encompassing diverse ecological and climatic zones; 23 of 47 counties were represented, and four nationwide studies were included.

In line with studies from other countries in Sub-Saharan African (SSA), literature from Kenya indicated decreasing population density to be linked to increasing snakebite risk [11,66], and rurality was cited as a risk factor for snakebite whenever measured [29–31,48–58,60–64]. Furthermore poverty indicators, such as housing constructed from earthen bricks, were associated with increased bite risk [29–31,48,49,51–57,61,62,64]. This supports findings from other regions with a stronger evidence base, such as South America and South Asia, where snakebite is reported as being a disease of poverty [3,25,29,30,49,51–55,57,61,62,64]. Only Larson et al., 2022 [50] did not find a link between lower SES and bite risk, instead they found rurality to be the strongest indicator for snakebite risk. However, these may not be independent variables as rurality and poverty metrics in Kenya are often linked [67].

Further aligning with literature from the broader global evidence base, bites were commonly reported to involve the lower limbs, linked to barefoot travel or minimal footwear during agricultural work [21,31,51,56–58,61,63]. Supporting this, walking and herding were commonly reported activities associated with increased snakebite risk, especially in savannah regions [29–31,48–58,60,61,63]. Savannah areas in Kenya were consistently associated with higher snakebite risk, this finding has implications for global snakebite risk models, particularly those that cover SSA, of which many do not include savannah as a risk habitat [24,68–70].

In Kenya the elevated risk in savannahs likely results from the spatial overlap of human activity and local terrestrial venomous snake species [29,39,48,50–56,58,60–64,67,71–73]. Kenya has many communities that practice pastoralism, and several medically important species from the Viperidae, Elapidae and Colubridae family that inhabit the savannah and cropland regions of Kenya [29]. Though two studies from ASAL regions did not find an increased risk of snakebite in cropland [49,56], cropland suitability for snakes may vary temporally and by crop type; for example, burning of sugar cane can temporarily reduce habitat suitability and snake presence [74], thereby potentially temporarily lowering bite incidence. Highlighting the risk of over generalising patterns when assessing snakebite prone landscapes.

This distinction may also be relevant for global snakebite risk models, where agricultural land is often associated with elevated risk [15,62], but where finer-scale information on crop type, burning regimes, crop cycles and seasonal land management could improve predictions. This may become increasingly important in rain-fed agricultural regions, where climate change is expected to alter rainfall regimes and agricultural adaptation strategies [75,76]. In turn, such changes to crop choice and cropping calendars have the potential to alter patterns of human–snake contact [69,75].

Evening and night-time exposure has been identified as an important component of snakebite risk in several settings, particularly in South Asia, where nocturnal bites from kraits (*Bungarus* spp.) are frequently reported indoors or while people are sleeping [14,21,22,77]. While some studies from Kenya supported this trend [29,51,52,56,57], several studies reported that bite risk was equally present, or in one instance higher, during the day [50,53,58,61]. These day time peaks may be associated with diurnal species presence, however due to unreliable species identification this was not possible to determine in this review. Day time peaks may also be influenced by occupational risks such as herding and farming [29,50,61,63]. In Kenya, as in many other SSA settings, participation in a range of occupations and livelihood activities is strongly gendered [78–80]. However, unlike other SSA countries [55], despite certain occupations carrying a higher inherent risk of snakebite the most common finding was no significant difference between sexes regarding snakebite incidence [29,31,50,55,57,58,61].

A recent study by Oluoch GO et al., 2025, published after this review’s search period ended, found similar health-seeking behaviour between men and women [32]. This suggests that the lack of sex differences in reported bite incidence in Kenya is unlikely to reflect women presenting to hospital more often than men. Instead, it supports the idea that snakebite risk in Kenya arises from multiple interacting social, environmental, and economic factors. Including gendered livelihoods, poverty, and the spatial overlap between people and snakes, highlighting the need for a One Health approach [49,50,56–58,63].

While globally the consensus is that human-snake conflict increases in the rainy season [10,12,25], half of all studies assessing seasonality in Kenya described either no seasonal difference in snake conflict [55,61] or higher conflict rates in the dry-season [49,50,58]. In-country experts hypothesised that increased dry-season bite risk may be driven by a combination of seasonal agricultural activities, consistent with evidence from Ghana [81], and increased human–snake contact around scarce water sources in very arid environments. However, these proposed mechanisms require further investigation.

Another theory around seasonality of bites comes from Mukeka 2019 [60] which noted that the overall peaks in human wildlife conflict (HWC) incidents corresponded with severe droughts, suggesting that extreme weather conditions can intensify HWC conflict [60]. The same study also cited an increase in HWC in areas where wildlife habitat is being converted to agricultural land [60], while a separate study from Kilifi County reported snakebite incidence rising in line with population growth [63]. These results underscore the need for strengthened prevention measures in urbanising locations [5]. They also highlight the importance of considering land use and climate change as drivers for future snake conflict, particularly in low- and-middle-income countries where snakebite remains a substantial public health concern [2,3,6].

### Methodological considerations and limitations of the study

Due to the low number of eligible studies, there was uneven county coverage (23/47 counties), and there was no study available on snakebite risk in purely non-ASAL counties. This has limited the conclusions that can be drawn about variable associations by geographic variation. A 2021 Health Action International (HAI) report noted substantially higher overall numbers of snakebites in ASAL than non-ASAL counties. However this link could not be investigated further due to the heterogeneity and limited studies available. A limitation of this study is the limited availability of unpublished and grey literature on snakebite in Kenya. Although findings were discussed with Kenyan governmental, academic and NGO partners, additional data were often unavailable or could not be shared, so the review relied primarily on published sources.

Time-bounded database screening may have missed additional grey literature. Accurate species identification in routine settings is often not feasible; we therefore did not assess outcomes by species. Heterogeneity of studies posed a challenge also, inconsistent definitions, measurements, and analytical approaches across studies precluded statistical pooling and led to a narrative synthesis of results. Many included studies were qualitative or retrospective, limiting causal inference and triangulation. Whilst several factors are consistently associated with snakebite risk across the counties, further studies would be needed to definitively prove the pattern of association between important environmental and demographic variables and risk. Thematic synthesis cannot yield pooled effect sizes, and conclusions from this review should be considered exploratory.

## Conclusion

Our findings challenge several global narratives about snakebite risk and emphasize the importance of local context. While globally other studies have found seasonality to be important, in Kenya, the seasonality of snakebite is not always reported to increase during the rainy season. In fact in some settings, snake conflict either shows no seasonal difference or peaks during the dry season, with reported seasonal patterns particularly varying in rural ASAL regions. However, the small sample size limits geographic conclusions that can be drawn and warrants further investigation. Temporal patterns also differ from global narrative, with some evidence of comparable or higher daytime bite incidence, particularly in northern ASAL counties. In other SSA countries there have been reported gender disparity in snakebite incidence, however we often found no clear gender disparity in snakebite risk. This suggests exposure is widespread across rural populations, especially within savannah ecosystems which support multiple medically important snake species.

Risk appears to arise from routine overlap between human and snake activity in shared landscapes, supporting a one health approach to mitigating snakebite incidence. Our findings reinforce the notion that snakebite is a disease of poverty in landscapes where daily exposure to snakes is more likely. Ongoing conversion of natural habitats to agricultural land may further intensify human–snake conflict. Given projections of substantial land-use change in Kenya over the coming decades, in line with many low- and middle-income in which snake bites are an issue, understanding how landscape transformation alters snakebite risk should be a priority for future research and public health policy planning. While reinforcing existing knowledge, our results highlight local nuances and the need for increased context-specific studies on snakebite risk.

## Supporting information

S1 PRISMA Checklist. Completed PRISMA 2020 checklist for the systematic review. The PRISMA 2020 checklist is reproduced under the Creative Commons Attribution 4.0 International (CC BY 4.0) licence from Page MJ, McKenzie JE, Bossuyt PM, et al. *The PRISMA 2020 statement: an updated guideline for reporting systematic reviews*. BMJ. 2021;372:n71.
(DOCX)

S1 Table Sources in languages other than English that progressed beyond title screening but did not meet the inclusion criteria. (DOCX)

S1 Fig Fisher’s exact test results for associations between reported snakebite risk factors. (TIF)

S2 Table Pairwise comparisons of snakebite risk factors. (DOCX)

S3 Table AACODS checklist guidelines used for quality assessment of grey literature. (PDF)

S4 Table Mixed Methods Appraisal Tool (MMAT) guidelines used for quality assessment of academic literature. (PDF)

S1 Data All risk factors identified during the initial search, including those reported in included studies and in additional studies not retained in the final review following PRISMA screening. (XLSX)

S5 Table Coding of risk factors from included academic and grey literature that passed quality and risk-of-bias assessment; raw data used for graphical visualisations generated in R. (CSV)
