# Supplementary material for: A narrative systematic review of snakebite risk factors in Kenya, incorporating grey and academic literature
Source: PLOS Glob Public Health. 2026 Oct 21;6(10):e0006344. doi: 10.1371/journal.pgph.0006344 (PMC13686475; doi:10.1371/journal.pgph.0006344)
Supplement: Supplementary file 4 [file pgph.0006344.s002.docx]

# Non-English Literature on Snakebite Risk Factors in Kenya: Evidence Map and Synthesis

## Executive summary

In Kenya specifically, the web-indexed, non-English document set that explicitly discusses risk factors (as opposed to only general background on snakebite) appears small and heavily skewed toward grey literature and journalism, rather than peer‑reviewed epidemiologic studies. PubMed, Scopus, Senate House Library and Google scholar were searched for non-English publications

Major gaps in the non-English Kenya‑specific literature are methodological: *few* accessible non-English documents provide (a) clear study designs, (b) sample sizes/denominators, (c) county-representative sampling frames, or (d) transparent measurement of exposure variables. By contrast, more rigorous risk‑factor epidemiology for Kenya appears to be published predominantly in English (not the focus of this evidence map).

## Search strategy and inclusion criteria

This review targeted academic papers and trusted grey literature discussing snakebite risk factors in Kenya, where the main text is not English (or the item is substantively non-English, even if metadata/title is English). Searches were run using multilingual terms for snakebite and risk (e.g., *morsure de serpent / facteurs de risque*; *Schlangenbiss / Risikofaktoren*; *kung’atwa na nyoka / hatari / kujikinga*), combined with Kenya country name (e.g., Kenia).

## Catalogue of non-English documents

The items below are the non-English documents found that explicitly describe snakebite risk factors in Kenya and are sufficiently attributable to identifiable sources. They are ordered to prioritize primary programmatic/official material first, then high-quality secondary reporting (because peer-reviewed Kenya risk-factor studies in non-English were rarely discoverable/accessible).

**Document A — NGO operational report (French)**
**Title (original):** *Rapport international d’activités 2019* (section: Kenya). [[8]](https://www.msf.org/sites/default/files/2020-10/MSF_Activity_Report_2019_FR_WEB_final.pdf)
**Title (English translation):** *International Activity Report 2019* (Kenya section). [[8]](https://www.msf.org/sites/default/files/2020-10/MSF_Activity_Report_2019_FR_WEB_final.pdf)
**Corporate author:** Médecins Sans Frontières[[9]](https://www.zeit.de/gesundheit/2023-07/schlangenbisse-kenia-schwarze-mamba-gegengift/seite-2). [[8]](https://www.msf.org/sites/default/files/2020-10/MSF_Activity_Report_2019_FR_WEB_final.pdf)
**Year:** Reporting year 2019 (publication file hosted with MSF activity report materials). [[8]](https://www.msf.org/sites/default/files/2020-10/MSF_Activity_Report_2019_FR_WEB_final.pdf)
**Language:** French. [[8]](https://www.msf.org/sites/default/files/2020-10/MSF_Activity_Report_2019_FR_WEB_final.pdf)
**Document type:** NGO annual activity report (programmatic narrative + operational highlights). [[8]](https://www.msf.org/sites/default/files/2020-10/MSF_Activity_Report_2019_FR_WEB_final.pdf)
**Geographic scope in Kenya:** Baringo County[[10]](https://www.zeit.de/gesundheit/2023-07/schlangenbisse-kenia-schwarze-mamba-gegengift/seite-2) (explicit). [[8]](https://www.msf.org/sites/default/files/2020-10/MSF_Activity_Report_2019_FR_WEB_final.pdf)
**Full text availability:** Yes (public PDF). [[8]](https://www.msf.org/sites/default/files/2020-10/MSF_Activity_Report_2019_FR_WEB_final.pdf)

**English summary (what it says about risk factors):**
The report describes a “high incidence” of snakebite in Baringo and frames an important *system risk factor* as underreporting, attributing it to insufficient monitoring/tracking tools. [[8]](https://www.msf.org/sites/default/files/2020-10/MSF_Activity_Report_2019_FR_WEB_final.pdf) It describes a response package that includes staff training in prevention strategies, antivenom administration, and first aid, and it notes augmentation of antivenom stocks—implicitly identifying health-worker training gaps and antivenom availability as determinants of outcomes. [[8]](https://www.msf.org/sites/default/files/2020-10/MSF_Activity_Report_2019_FR_WEB_final.pdf)

**Extracted risk factors (explicit/implicit, Kenya-specific):**

- **High local incidence** in Baringo (contextual exposure burden). [[8]](https://www.msf.org/sites/default/files/2020-10/MSF_Activity_Report_2019_FR_WEB_final.pdf)
- **Underreporting** driven by **lack of appropriate monitoring tools** (surveillance/system risk factor). [[8]](https://www.msf.org/sites/default/files/2020-10/MSF_Activity_Report_2019_FR_WEB_final.pdf)
- **Insufficient health-worker preparedness** (training needed for prevention, first aid, and antivenom administration). [[8]](https://www.msf.org/sites/default/files/2020-10/MSF_Activity_Report_2019_FR_WEB_final.pdf)
- **Antivenom supply constraints** (report notes provision of vials to increase county stocks). [[8]](https://www.msf.org/sites/default/files/2020-10/MSF_Activity_Report_2019_FR_WEB_final.pdf)

**Not included because:**

- Not risk factors but snakebite occuring in the first instance, health care systems are a a risk to outcome of bite not bite incidence itself.

**Document B — Community newsletter prevention messaging (Swahili content)**
**Title (as posted):** *TIST Kenya Newsletter - June 2023*. [[3]](https://news.tist.org/web/node/391)
**Title (English translation):** Same as posted (title is English; content excerpt is Swahili). [[3]](https://news.tist.org/web/node/391)
**Publisher:** The International Small Group and Tree Planting Program[[11]](https://www.cnews.fr/monde/2019-03-19/morsures-de-serpents-un-poison-toujours-aussi-meurtrier-822494) (TIST). [[3]](https://news.tist.org/web/node/391)
**Year:** 2023 (June newsletter). [[3]](https://news.tist.org/web/node/391)
**Language:** Swahili (risk-related section in Swahili). [[3]](https://news.tist.org/web/node/391)
**Document type:** Newsletter / safety and community education message (grey literature). [[3]](https://news.tist.org/web/node/391)
**Geographic scope in Kenya:** Kenya (newsletter branded for Kenya; not county-specific in excerpt). [[3]](https://news.tist.org/web/node/391)
**Full text availability:** Yes (web page). [[3]](https://news.tist.org/web/node/391)

**English summary (what it says about risk factors):**
The newsletter provides prevention guidance that operationalizes exposure risk: it warns against moving through areas where you cannot see foot placement and recommends using a stick to sweep through brush/grass to detect snakes, implying that poor visibility and contact with vegetation elevate encounter risk. [[3]](https://news.tist.org/web/node/391) It also emphasizes that there is “no first aid” substitute and urges prompt hospital care, indirectly linking outcomes to rapid access to formal treatment. [[3]](https://news.tist.org/web/node/391)

**Extracted risk factors (explicit/implicit, Kenya-specific):**

- **Poor visibility during movement** (cannot see where you step). [[3]](https://news.tist.org/web/node/391)
- **Dense vegetation/brush/grass** as encounter environments. [[3]](https://news.tist.org/web/node/391)
- **Delayed presentation to hospital** as an outcome risk (prevention messaging stresses immediacy). [[3]](https://news.tist.org/web/node/391)

**Not included because:**

- The newsletter NGO is not from established herpetologists, Non-Government organisations (NGOs) with a specialism in snakebite, or government databases and reports, and does not specify whether guidance is based on local surveillance or general best practice. [[3]](https://news.tist.org/web/node/391)

**Document C — Government-linked broadcast item (Swahili)**
**Title (original, Swahili headline):** *Hospitali 86 zapokea dawa za kukabiliana na sumu ya nyoka Kitui* (KBC video page). [[12]](https://www.kenyamoja.com/video/hospitali-86-zapokea-dawa-za-kukabiliana-na-sumu-ya-nyoka-kitui-kbc-video-19250)
**Title (English translation):** *Eighty-six hospitals/health facilities receive medicine to counter snake venom in Kitui.* [[12]](https://www.kenyamoja.com/video/hospitali-86-zapokea-dawa-za-kukabiliana-na-sumu-ya-nyoka-kitui-kbc-video-19250)
**Publisher/broadcaster:** Kenyan Broadcasting Corporation[[13]](https://news.tist.org/web/node/391) (KBC). [[12]](https://www.kenyamoja.com/video/hospitali-86-zapokea-dawa-za-kukabiliana-na-sumu-ya-nyoka-kitui-kbc-video-19250)
**Year:** 2025 (page date shown). [[12]](https://www.kenyamoja.com/video/hospitali-86-zapokea-dawa-za-kukabiliana-na-sumu-ya-nyoka-kitui-kbc-video-19250)
**Language:** Swahili. [[12]](https://www.kenyamoja.com/video/hospitali-86-zapokea-dawa-za-kukabiliana-na-sumu-ya-nyoka-kitui-kbc-video-19250)
**Document type:** Broadcast news video page (government-linked public communication; grey literature adjacent). [[12]](https://www.kenyamoja.com/video/hospitali-86-zapokea-dawa-za-kukabiliana-na-sumu-ya-nyoka-kitui-kbc-video-19250)
**Geographic scope in Kenya:** Kitui County[[14]](https://news.tist.org/web/node/391) (explicit). [[12]](https://www.kenyamoja.com/video/hospitali-86-zapokea-dawa-za-kukabiliana-na-sumu-ya-nyoka-kitui-kbc-video-19250)
**Full text availability:** Partial (short video page + summary; full spoken content not transcribed in the excerpt). [[12]](https://www.kenyamoja.com/video/hospitali-86-zapokea-dawa-za-kukabiliana-na-sumu-ya-nyoka-kitui-kbc-video-19250)

**English summary (what it says about risk factors):**
The item reports that the health ministry sent antivenom/medicine to 86 facilities in Kitui to respond to an **increase in snakebite cases**, which functions as a signal of localized risk burden. [[12]](https://www.kenyamoja.com/video/hospitali-86-zapokea-dawa-za-kukabiliana-na-sumu-ya-nyoka-kitui-kbc-video-19250) It primarily documents a **response measure** (supply distribution), rather than analyzing ecological or occupational determinants.

**Extracted risk factors (explicit/implicit, Kenya-specific):**

- **Rising incidence / increased case burden** in Kitui (as the stated rationale for antivenom distribution). [[12]](https://www.kenyamoja.com/video/hospitali-86-zapokea-dawa-za-kukabiliana-na-sumu-ya-nyoka-kitui-kbc-video-19250)
- **Facility readiness / stock availability** as an outcome determinant (implied by distribution to facilities). [[12]](https://www.kenyamoja.com/video/hospitali-86-zapokea-dawa-za-kukabiliana-na-sumu-ya-nyoka-kitui-kbc-video-19250)

**Not included because:**

- Report focuses on afterbite vulnerabilities such as antivenom availability, not risk factors affecting bite risk occurrence.

**Document D — Media report emphasizing access delays and antivenom stockouts (French)**
**Title (original):** *Morsures de serpents : un poison toujours aussi meurtrier* (CNews). [[5]](https://www.cnews.fr/monde/2019-03-19/morsures-de-serpents-un-poison-toujours-aussi-meurtrier-822494)
**Title (English translation):** *Snakebites: a poison still just as deadly.* [[5]](https://www.cnews.fr/monde/2019-03-19/morsures-de-serpents-un-poison-toujours-aussi-meurtrier-822494)
**Publisher:** CNews[[15]](https://www.msf.org/sites/default/files/2020-10/MSF_Activity_Report_2019_FR_WEB_final.pdf). [[5]](https://www.cnews.fr/monde/2019-03-19/morsures-de-serpents-un-poison-toujours-aussi-meurtrier-822494)
**Year:** 2019 (page date shown). [[5]](https://www.cnews.fr/monde/2019-03-19/morsures-de-serpents-un-poison-toujours-aussi-meurtrier-822494)
**Language:** French. [[5]](https://www.cnews.fr/monde/2019-03-19/morsures-de-serpents-un-poison-toujours-aussi-meurtrier-822494)
**Document type:** News report (secondary reporting; not peer-reviewed). [[5]](https://www.cnews.fr/monde/2019-03-19/morsures-de-serpents-un-poison-toujours-aussi-meurtrier-822494)
**Geographic scope in Kenya:** Lake Baringo area with referrals through facilities including Marigat[[16]](https://www.cnews.fr/monde/2019-03-19/morsures-de-serpents-un-poison-toujours-aussi-meurtrier-822494), Kabarnet[[17]](https://news.tist.org/web/node/391), and Eldoret[[18]](https://news.tist.org/web/node/391). [[5]](https://www.cnews.fr/monde/2019-03-19/morsures-de-serpents-un-poison-toujours-aussi-meurtrier-822494)
**Full text availability:** Yes (article page). [[5]](https://www.cnews.fr/monde/2019-03-19/morsures-de-serpents-un-poison-toujours-aussi-meurtrier-822494)

**English summary (what it says about risk factors):**
The report narrates a case in which night-time transport delays (finding a motorcycle taxi) and multi-step referral interacted with antivenom stockout at one facility, requiring transfer to another hospital farther away. [[5]](https://www.cnews.fr/monde/2019-03-19/morsures-de-serpents-un-poison-toujours-aussi-meurtrier-822494) These details illustrate risk factors for severe outcomes: distance to definitive care, time-to-treatment, and antivenom availability. [[5]](https://www.cnews.fr/monde/2019-03-19/morsures-de-serpents-un-poison-toujours-aussi-meurtrier-822494)

**Extracted risk factors (explicit/implicit, Kenya-specific):**

- **Night travel / transport friction** delaying arrival at care. [[5]](https://www.cnews.fr/monde/2019-03-19/morsures-de-serpents-un-poison-toujours-aussi-meurtrier-822494)
- **Referral chain complexity** (multiple facilities before reaching antivenom-equipped care). [[5]](https://www.cnews.fr/monde/2019-03-19/morsures-de-serpents-un-poison-toujours-aussi-meurtrier-822494)
- **Antivenom stockout** at a local hospital (Kabarnet in the narrative). [[5]](https://www.cnews.fr/monde/2019-03-19/morsures-de-serpents-un-poison-toujours-aussi-meurtrier-822494)
- **Distance to regional referral hospital** (Eldoret in the narrative). [[5]](https://www.cnews.fr/monde/2019-03-19/morsures-de-serpents-un-poison-toujours-aussi-meurtrier-822494)

**Not included because:**

- Report focuses on afterbite vulnerabilities such as antivenom availability, not risk factors affecting bite risk occurrence.

**Document E — Media interview emphasizing behavioral and system risk factors (German)**
**Title (original):** *Schlangenbisse in Kenia: "Die Situation ist katastrophal"* (ZEIT ONLINE interview; excerpted). [[19]](https://www.zeit.de/gesundheit/2023-07/schlangenbisse-kenia-schwarze-mamba-gegengift)
**Title (English translation):** *Snakebites in Kenya: “The situation is catastrophic.”* [[19]](https://www.zeit.de/gesundheit/2023-07/schlangenbisse-kenia-schwarze-mamba-gegengift)
**Publisher:** Die Zeit[[20]](https://www.msf.org/sites/default/files/2020-10/MSF_Activity_Report_2019_FR_WEB_final.pdf). [[19]](https://www.zeit.de/gesundheit/2023-07/schlangenbisse-kenia-schwarze-mamba-gegengift)
**Year:** 2023 (July date shown). [[19]](https://www.zeit.de/gesundheit/2023-07/schlangenbisse-kenia-schwarze-mamba-gegengift)
**Language:** German. [[19]](https://www.zeit.de/gesundheit/2023-07/schlangenbisse-kenia-schwarze-mamba-gegengift)
**Document type:** Interview (secondary reporting). [[19]](https://www.zeit.de/gesundheit/2023-07/schlangenbisse-kenia-schwarze-mamba-gegengift)
**Geographic scope in Kenya:** Includes discussion of planned production in Nairobi[[21]](https://www.deutschlandfunkkultur.de/schlangenbisse-in-kenia-toedlich-aber-nicht-lukrativ-dlf-kultur-0a011926-100.html) and rural areas “far from major cities.” [[7]](https://www.zeit.de/gesundheit/2023-07/schlangenbisse-kenia-schwarze-mamba-gegengift/seite-2)
**Full text availability:** Mixed (paywall indicated; excerpt viewable via search snippet). [[19]](https://www.zeit.de/gesundheit/2023-07/schlangenbisse-kenia-schwarze-mamba-gegengift)

**English summary (what it says about risk factors):**
The interview emphasizes that insufficient antivenom production and knowledge of need drives preventable deaths, and it notes the need to know seasonality and frequency of bites to plan supply. [[22]](https://www.zeit.de/gesundheit/2023-07/schlangenbisse-kenia-schwarze-mamba-gegengift/seite-2) It also highlights care-seeking behavior: people far from cities may visit traditional healers (example given: a “black stone” applied to the wound), which can delay biomedical treatment. [[7]](https://www.zeit.de/gesundheit/2023-07/schlangenbisse-kenia-schwarze-mamba-gegengift/seite-2) It finally points to practical household/community prevention measures (e.g., lighting and bed nets) and the importance of minimizing risky encounters by respecting snake habitats. [[7]](https://www.zeit.de/gesundheit/2023-07/schlangenbisse-kenia-schwarze-mamba-gegengift/seite-2)

**Extracted risk factors (explicit/implicit, Kenya-specific):**

- **Antivenom scarcity and supply-chain limitations** (system risk factor). [[19]](https://www.zeit.de/gesundheit/2023-07/schlangenbisse-kenia-schwarze-mamba-gegengift)
- **Insufficient surveillance/knowledge of demand** (need seasonal and incidence data). [[7]](https://www.zeit.de/gesundheit/2023-07/schlangenbisse-kenia-schwarze-mamba-gegengift/seite-2)
- **Distance from major cities** (access barrier). [[7]](https://www.zeit.de/gesundheit/2023-07/schlangenbisse-kenia-schwarze-mamba-gegengift/seite-2)
- **Traditional healer use delaying formal care** (behavioral pathway). [[7]](https://www.zeit.de/gesundheit/2023-07/schlangenbisse-kenia-schwarze-mamba-gegengift/seite-2)
- **Household/community prevention gaps** (need for lighting, protective sleeping arrangements, and awareness). [[7]](https://www.zeit.de/gesundheit/2023-07/schlangenbisse-kenia-schwarze-mamba-gegengift/seite-2)

**Not included because:**

- The newsletter NGO is not from established herpetologists, Non-Government organisations (NGOs) with a specialism in snakebite, or government databases and reports, and does not specify whether guidance is based on local surveillance or general best practice. [[3]](https://news.tist.org/web/node/391)

**Document F — Media report emphasizing antivenom production gap (German)**
**Title (original):** *Schlangenbisse in Kenia – Afrika braucht eigenes Gegengift* (Deutschlandfunk Kultur). [[23]](https://www.deutschlandfunkkultur.de/schlangenbisse-in-kenia-toedlich-aber-nicht-lukrativ-dlf-kultur-0a011926-100.html)
**Title (English translation):** *Snakebites in Kenya – Africa needs its own antidote.* [[23]](https://www.deutschlandfunkkultur.de/schlangenbisse-in-kenia-toedlich-aber-nicht-lukrativ-dlf-kultur-0a011926-100.html)
**Publisher:** Deutschlandfunk Kultur[[24]](https://news.tist.org/web/node/391). [[23]](https://www.deutschlandfunkkultur.de/schlangenbisse-in-kenia-toedlich-aber-nicht-lukrativ-dlf-kultur-0a011926-100.html)
**Year:** 2023 (July date shown). [[23]](https://www.deutschlandfunkkultur.de/schlangenbisse-in-kenia-toedlich-aber-nicht-lukrativ-dlf-kultur-0a011926-100.html)
**Language:** German. [[23]](https://www.deutschlandfunkkultur.de/schlangenbisse-in-kenia-toedlich-aber-nicht-lukrativ-dlf-kultur-0a011926-100.html)
**Document type:** Radio/podcast journalism. [[23]](https://www.deutschlandfunkkultur.de/schlangenbisse-in-kenia-toedlich-aber-nicht-lukrativ-dlf-kultur-0a011926-100.html)
**Geographic scope in Kenya:** Kenya (national framing). [[23]](https://www.deutschlandfunkkultur.de/schlangenbisse-in-kenia-toedlich-aber-nicht-lukrativ-dlf-kultur-0a011926-100.html)
**Full text availability:** Page accessible; audio referenced. [[23]](https://www.deutschlandfunkkultur.de/schlangenbisse-in-kenia-toedlich-aber-nicht-lukrativ-dlf-kultur-0a011926-100.html)

**English summary (what it says about risk factors):**
The piece frames the core outcome risk as driven by insufficient antivenom supply, describing an initiative to establish local production capacity in Kenya. [[23]](https://www.deutschlandfunkkultur.de/schlangenbisse-in-kenia-toedlich-aber-nicht-lukrativ-dlf-kultur-0a011926-100.html) This aligns with other Kenya-focused sources emphasizing antivenom scarcity as a determinant of mortality and disability.

**Extracted risk factors (explicit/implicit, Kenya-specific):**

- **Antivenom availability and production constraints** affecting outcomes. [[23]](https://www.deutschlandfunkkultur.de/schlangenbisse-in-kenia-toedlich-aber-nicht-lukrativ-dlf-kultur-0a011926-100.html)

**Not included because:**

- Report focuses on afterbite vulnerabilities such as antivenom availability, not risk factors affecting bite risk occurrence.
- The newsletter NGO is not from established herpetologists, Non-Government organisations (NGOs) with a specialism in snakebite, or government databases and reports, and does not specify whether guidance is based on local surveillance or general best practice. [[3]](https://news.tist.org/web/node/391)

## Comparative table of documents

| ID | Language | Document type | Kenya geographic specificity | Risk factors explicitly discussed (Kenya-linked) | Methods / quality indicators (what can be extracted) | Full-text access |
| --- | --- | --- | --- | --- | --- | --- |
| A | French | NGO annual activity report | Baringo County | High incidence; underreporting due to inadequate tracking tools; training gaps; antivenom stock constraints | Program narrative; no denominator/sample; operational description of training and antivenom provision | Public PDF [[8]](https://www.msf.org/sites/default/files/2020-10/MSF_Activity_Report_2019_FR_WEB_final.pdf) |
| B | Swahili (section content) | NGO/community newsletter | Kenya (general) | Poor visibility/foot placement; brush/grass exposure; urgency of hospital care | Educational guidance; no evaluation design | Public web page [[3]](https://news.tist.org/web/node/391) |
| C | Swahili | Broadcast news item | Kitui County | Increased cases prompting distribution; facility stock readiness | News summary; no rates/denominator; limited methodological detail | Public video page/summary [[12]](https://www.kenyamoja.com/video/hospitali-86-zapokea-dawa-za-kukabiliana-na-sumu-ya-nyoka-kitui-kbc-video-19250) |
| D | French | News report (case narrative) | Lake Baringo referral pathway; Marigat → Kabarnet → Eldoret | Night-time access delays; referral chain; antivenom stockout; long-distance transfer | Case narrative; not generalizable; illustrates mechanisms rather than quantifying them | Public article page [[5]](https://www.cnews.fr/monde/2019-03-19/morsures-de-serpents-un-poison-toujours-aussi-meurtrier-822494) |
| E | German | Interview journalism | Nairobi + rural access framing | Antivenom scarcity; lack of seasonal/incidence data; rural distance barriers; traditional healer use; prevention measures (lighting/bed nets) | Interview-based; perspective-driven; partially paywalled | Partial/paywalled [[19]](https://www.zeit.de/gesundheit/2023-07/schlangenbisse-kenia-schwarze-mamba-gegengift) |
| F | German | Public radio/podcast journalism | Kenya (national framing) | Antivenom production/supply gap as outcome risk | Journalism; no sampling | Public page/audio [[23]](https://www.deutschlandfunkkultur.de/schlangenbisse-in-kenia-toedlich-aber-nicht-lukrativ-dlf-kultur-0a011926-100.html) |

## Synthesis of risk factors and evidence gaps

### Common risk factors across non-English sources

**Environmental and activity-driven exposure (encounter probability)**

- **Low-visibility movement and contact with vegetation** emerges clearly in Swahili prevention messaging: walking where you cannot see foot placement and moving through grass/brush are framed as bite-risk contexts. [[3]](https://news.tist.org/web/node/391)
- Several Kenya-focused narratives indirectly situate risk in **rural environments** where daily life involves outdoor movement and where snakes may be encountered near homes or paths; this is reinforced by global WHO framing that rural and agricultural populations are often most affected. [[25]](https://www.who.int/es/news-room/fact-sheets/detail/snakebite-envenoming)

**Access and treatment pathway risks (severity, disability, mortality)**

- **Antivenom availability** is the most consistently repeated Kenya-linked system risk factor: MSF reports supplementing stocks and building staff capacity; media pieces highlight stockouts and the need for local production. [[26]](https://www.msf.org/sites/default/files/2020-10/MSF_Activity_Report_2019_FR_WEB_final.pdf)
- **Time-to-treatment delays** appear as practical risk amplifiers through night-time transport delay and multi-step referral in county settings. [[5]](https://www.cnews.fr/monde/2019-03-19/morsures-de-serpents-un-poison-toujours-aussi-meurtrier-822494)
- **Surveillance and data gaps** (monitoring tools, capturing seasonal patterns) are repeatedly framed as barriers to planning prevention and antivenom supply—an upstream risk factor shaping downstream outcomes. [[6]](https://www.msf.org/sites/default/files/2020-10/MSF_Activity_Report_2019_FR_WEB_final.pdf)

**Care-seeking behavior and sociocultural determinants**

- The German interview excerpt explicitly points to **traditional healer use** among populations far from cities (example practice described) as a pathway to delayed biomedical care. [[7]](https://www.zeit.de/gesundheit/2023-07/schlangenbisse-kenia-schwarze-mamba-gegengift/seite-2)

### Gaps specific to non-English Kenya literature

The most striking gap is the **lack of non-English primary epidemiologic studies** on Kenya risk factors (with defined sampling frames, measured exposures, and multivariable analysis). In the retrievable non-English set, Kenya-specific risk factors are largely captured through:

- Programmatic reporting describing operational barriers (monitoring tools, training, supply). [[8]](https://www.msf.org/sites/default/files/2020-10/MSF_Activity_Report_2019_FR_WEB_final.pdf)
- Prevention messaging translating general encounter-risk logic into Swahili guidance (visibility, vegetation). [[3]](https://news.tist.org/web/node/391)
- Journalism documenting access barriers and stockouts as lived experience rather than measured predictors. [[27]](https://www.cnews.fr/monde/2019-03-19/morsures-de-serpents-un-poison-toujours-aussi-meurtrier-822494)

As a result, the non-English corpus provides **credible hypotheses** about risk mechanisms and system bottlenecks, but offers **limited quantification** of risk factor strength, interaction (e.g., seasonality × occupation × distance), or comparability.

## Mermaid relationship map

```
flowchart LR
  RF1[Environmental exposure:\nlow visibility, brush/grass, foot placement] --> B[TIST Kenya Newsletter (Swahili)\nJune 2023]
  RF2[Health-system:\nantivenom stock & availability] --> A[MSF International Activity Report 2019 (French)]
  RF2 --> D[CNews report (French)\nBaringo referral delays & stockout]
  RF2 --> E[Die Zeit interview (German)\nantivenom scarcity & production]
  RF2 --> F[Deutschlandfunk Kultur (German)\nneed for local antidote]
  RF3[Access:\nnight transport, referral pathways] --> D
  RF4[Surveillance:\nunderreporting, weak monitoring tools,\nunknown seasonality & demand] --> A
  RF4 --> E
  RF5[Care-seeking behavior:\ntraditional healer use delaying care] --> E
  RF6[Signal of localized burden:\nreported increase in cases] --> C[KBC report (Swahili)\nKitui distribution to 86 facilities]
```

[[1]](https://www.who.int/es/news-room/fact-sheets/detail/snakebite-envenoming) [[25]](https://www.who.int/es/news-room/fact-sheets/detail/snakebite-envenoming) https://www.who.int/es/news-room/fact-sheets/detail/snakebite-envenoming

<https://www.who.int/es/news-room/fact-sheets/detail/snakebite-envenoming>

[[2]](https://www.msf.org/sites/default/files/2020-10/MSF_Activity_Report_2019_FR_WEB_final.pdf) [[4]](https://www.msf.org/sites/default/files/2020-10/MSF_Activity_Report_2019_FR_WEB_final.pdf) [[6]](https://www.msf.org/sites/default/files/2020-10/MSF_Activity_Report_2019_FR_WEB_final.pdf) [[8]](https://www.msf.org/sites/default/files/2020-10/MSF_Activity_Report_2019_FR_WEB_final.pdf) [[15]](https://www.msf.org/sites/default/files/2020-10/MSF_Activity_Report_2019_FR_WEB_final.pdf) [[20]](https://www.msf.org/sites/default/files/2020-10/MSF_Activity_Report_2019_FR_WEB_final.pdf) [[26]](https://www.msf.org/sites/default/files/2020-10/MSF_Activity_Report_2019_FR_WEB_final.pdf) https://www.msf.org/sites/default/files/2020-10/MSF_Activity_Report_2019_FR_WEB_final.pdf

<https://www.msf.org/sites/default/files/2020-10/MSF_Activity_Report_2019_FR_WEB_final.pdf>

[[3]](https://news.tist.org/web/node/391) [[13]](https://news.tist.org/web/node/391) [[14]](https://news.tist.org/web/node/391) [[17]](https://news.tist.org/web/node/391) [[18]](https://news.tist.org/web/node/391) [[24]](https://news.tist.org/web/node/391) https://news.tist.org/web/node/391

<https://news.tist.org/web/node/391>

[[5]](https://www.cnews.fr/monde/2019-03-19/morsures-de-serpents-un-poison-toujours-aussi-meurtrier-822494) [[11]](https://www.cnews.fr/monde/2019-03-19/morsures-de-serpents-un-poison-toujours-aussi-meurtrier-822494) [[16]](https://www.cnews.fr/monde/2019-03-19/morsures-de-serpents-un-poison-toujours-aussi-meurtrier-822494) [[27]](https://www.cnews.fr/monde/2019-03-19/morsures-de-serpents-un-poison-toujours-aussi-meurtrier-822494) https://www.cnews.fr/monde/2019-03-19/morsures-de-serpents-un-poison-toujours-aussi-meurtrier-822494

<https://www.cnews.fr/monde/2019-03-19/morsures-de-serpents-un-poison-toujours-aussi-meurtrier-822494>

[[7]](https://www.zeit.de/gesundheit/2023-07/schlangenbisse-kenia-schwarze-mamba-gegengift/seite-2) [[9]](https://www.zeit.de/gesundheit/2023-07/schlangenbisse-kenia-schwarze-mamba-gegengift/seite-2) [[10]](https://www.zeit.de/gesundheit/2023-07/schlangenbisse-kenia-schwarze-mamba-gegengift/seite-2) [[22]](https://www.zeit.de/gesundheit/2023-07/schlangenbisse-kenia-schwarze-mamba-gegengift/seite-2) https://www.zeit.de/gesundheit/2023-07/schlangenbisse-kenia-schwarze-mamba-gegengift/seite-2

<https://www.zeit.de/gesundheit/2023-07/schlangenbisse-kenia-schwarze-mamba-gegengift/seite-2>

[[12]](https://www.kenyamoja.com/video/hospitali-86-zapokea-dawa-za-kukabiliana-na-sumu-ya-nyoka-kitui-kbc-video-19250) https://www.kenyamoja.com/video/hospitali-86-zapokea-dawa-za-kukabiliana-na-sumu-ya-nyoka-kitui-kbc-video-19250

<https://www.kenyamoja.com/video/hospitali-86-zapokea-dawa-za-kukabiliana-na-sumu-ya-nyoka-kitui-kbc-video-19250>

[[19]](https://www.zeit.de/gesundheit/2023-07/schlangenbisse-kenia-schwarze-mamba-gegengift) https://www.zeit.de/gesundheit/2023-07/schlangenbisse-kenia-schwarze-mamba-gegengift

<https://www.zeit.de/gesundheit/2023-07/schlangenbisse-kenia-schwarze-mamba-gegengift>

[[21]](https://www.deutschlandfunkkultur.de/schlangenbisse-in-kenia-toedlich-aber-nicht-lukrativ-dlf-kultur-0a011926-100.html) [[23]](https://www.deutschlandfunkkultur.de/schlangenbisse-in-kenia-toedlich-aber-nicht-lukrativ-dlf-kultur-0a011926-100.html) https://www.deutschlandfunkkultur.de/schlangenbisse-in-kenia-toedlich-aber-nicht-lukrativ-dlf-kultur-0a011926-100.html

<https://www.deutschlandfunkkultur.de/schlangenbisse-in-kenia-toedlich-aber-nicht-lukrativ-dlf-kultur-0a011926-100.html>
