# Supplementary material for: A narrative systematic review of snakebite risk factors in Kenya, incorporating grey and academic literature
Source: PLOS Glob Public Health. 2026 Oct 21;6(10):e0006344. doi: 10.1371/journal.pgph.0006344 (PMC13686475; doi:10.1371/journal.pgph.0006344)
Supplement: Supplementary file 5 [file pgph.0006344.s004.docx]

**Figure 2. Pairwise comparison between selection of results:**

Across head-to-head sign tests, only time of day showed a consistent direction. Night vs day: In 12 studies that reported both, 8 favoured higher risk at night and none favoured day (4 ties); this was significant and remained so after FDR correction (*p* = 0.008; q = 0.047), indicating higher snakebite risk at night. Age: Among 14 studies, over-18 was favoured in 5 vs 0 for under-18 (9 ties), but this did not reach significance (q = 0.188). Bite site: Of 8 studies, foot/lower limb was favoured in 3 vs 0 for arm/upper limb (5 ties); not significant. Season: In 10 studies, rainy vs dry was split (5 vs 3; 2 ties); not significant. Location (activity setting): In 16 studies, outdoors was favoured in 3 vs 1 for indoors (12 ties); not significant. Sex: In 14 studies, results were near null (male 2 vs female 1; 11 ties); not significant. Overall, aside from a night-time elevation in risk, other contrasts showed no clear, consistent direction, with many ties and small numbers, consistent with heterogeneity across study designs and settings**.**

| **Pair** | **n compared** | **A wins** | **B wins** | **Ties** | **p (binomial)** | **p (FDR)** | **Sig** | **Direction** |  |
| --- | --- | --- | --- | --- | --- | --- | --- | --- | --- |
| **Day Time vs Night Time** | **12** | **0** | **8** | **4** | **0.008** | **0.047** | ***** | **Night Time > Day Time (consistent)** | |
| **Under 18 (Risk/Incidence) vs Over 18 (Risk/Incidence)** | **14** | **0** | **5** | **9** | **0.062** | **0.188** |  | **No clear direction** | |
| **Bite Location (Foot/ Lower Limb) vs Bite Location (Arm/ Upper Limb)** | **8** | **3** | **0** | **5** | **0.25** | **0.5** |  | **No clear direction** | |
| **Rainy Season vs Dry Season** | **10** | **5** | **3** | **2** | **0.727** | **0.872** |  | **No clear direction** | |
| **Indoors (Location) vs Outdoors (Location)** | **16** | **1** | **3** | **12** | **0.625** | **0.872** |  | **No clear direction** | |
| **Male (Higher Incidence/Risk) vs Female (Higher Incidence/Risk)** | **14** | **2** | **1** | **11** | **1** | **1** |  | **No clear direction** | |
|  |  |  |  |  |  |  |  |  | |
|  |  |  |  |  |  |  |  |  | |
